# Supplementary figures and images for: Members of the abscisic acid co‐receptor PP2C protein family mediate salicylic acid–abscisic acid crosstalk
Source: Plant Direct. 2017 Nov 6;1(5):e00020. doi: 10.1002/pld3.20 (PMC6508495; doi:10.1002/pld3.20)

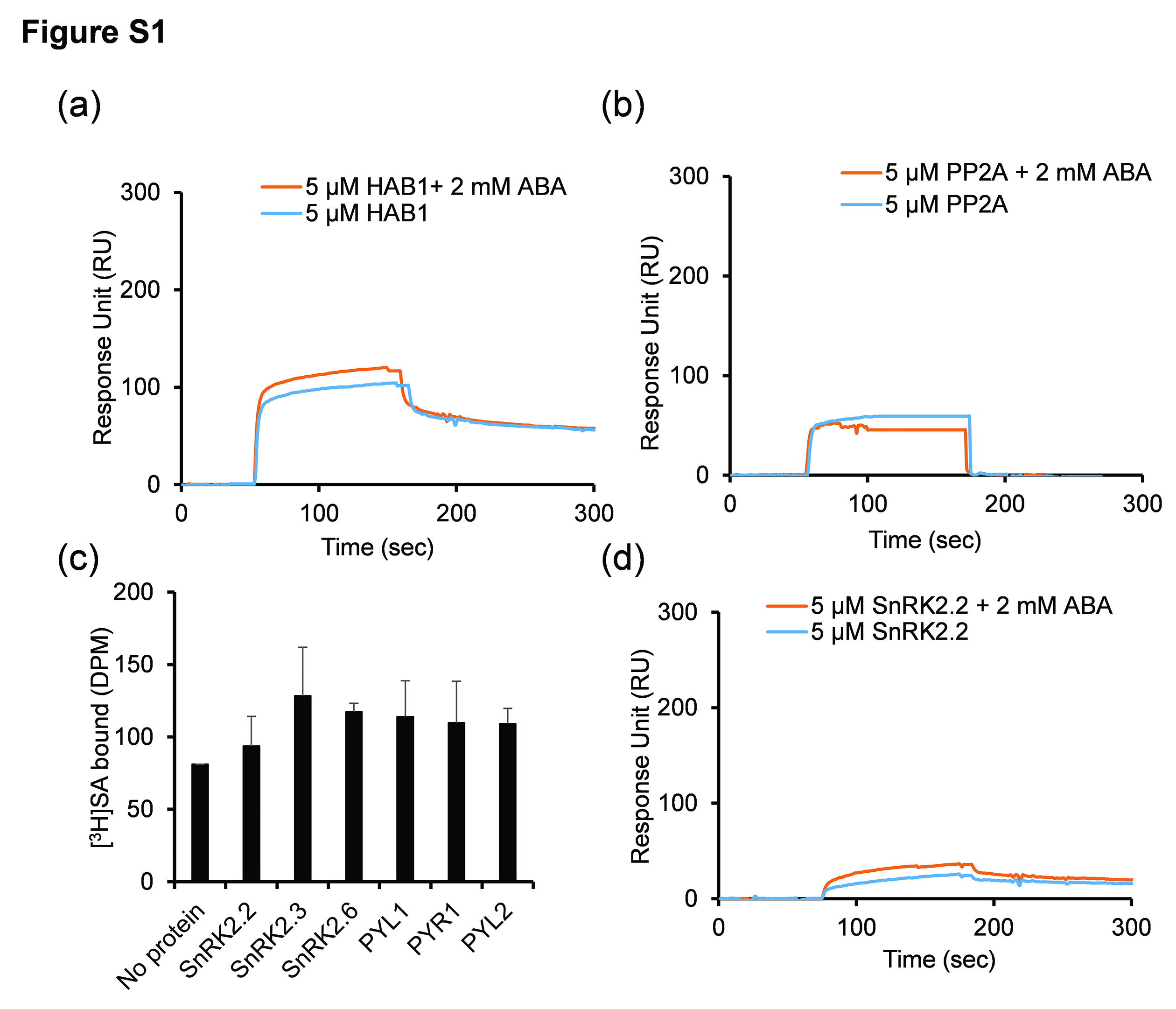

Supplement: Supplementary file 1 [file PLD3-1-e00020-s007.tif]

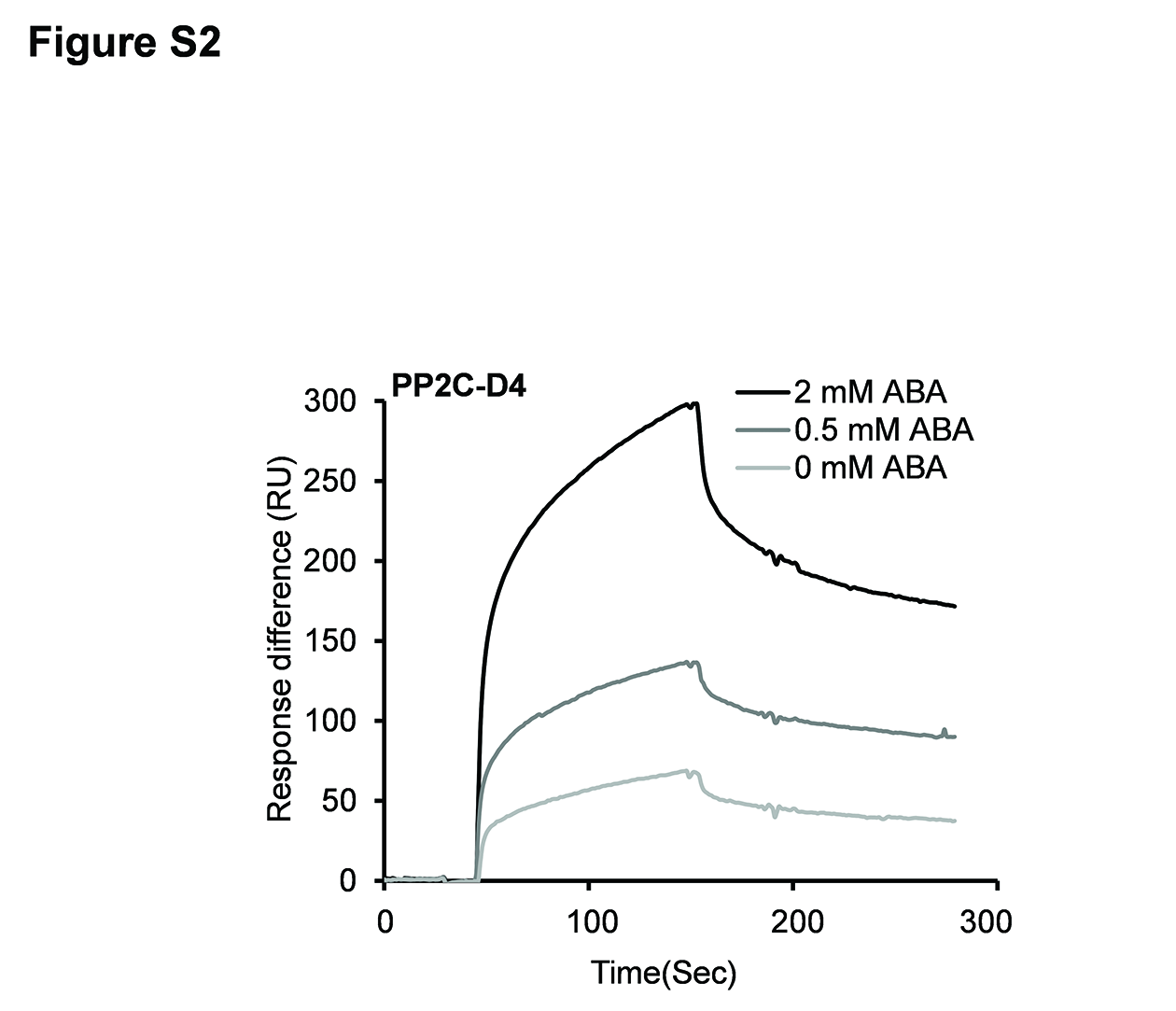

Supplement: Supplementary file 2 [file PLD3-1-e00020-s001.tif]

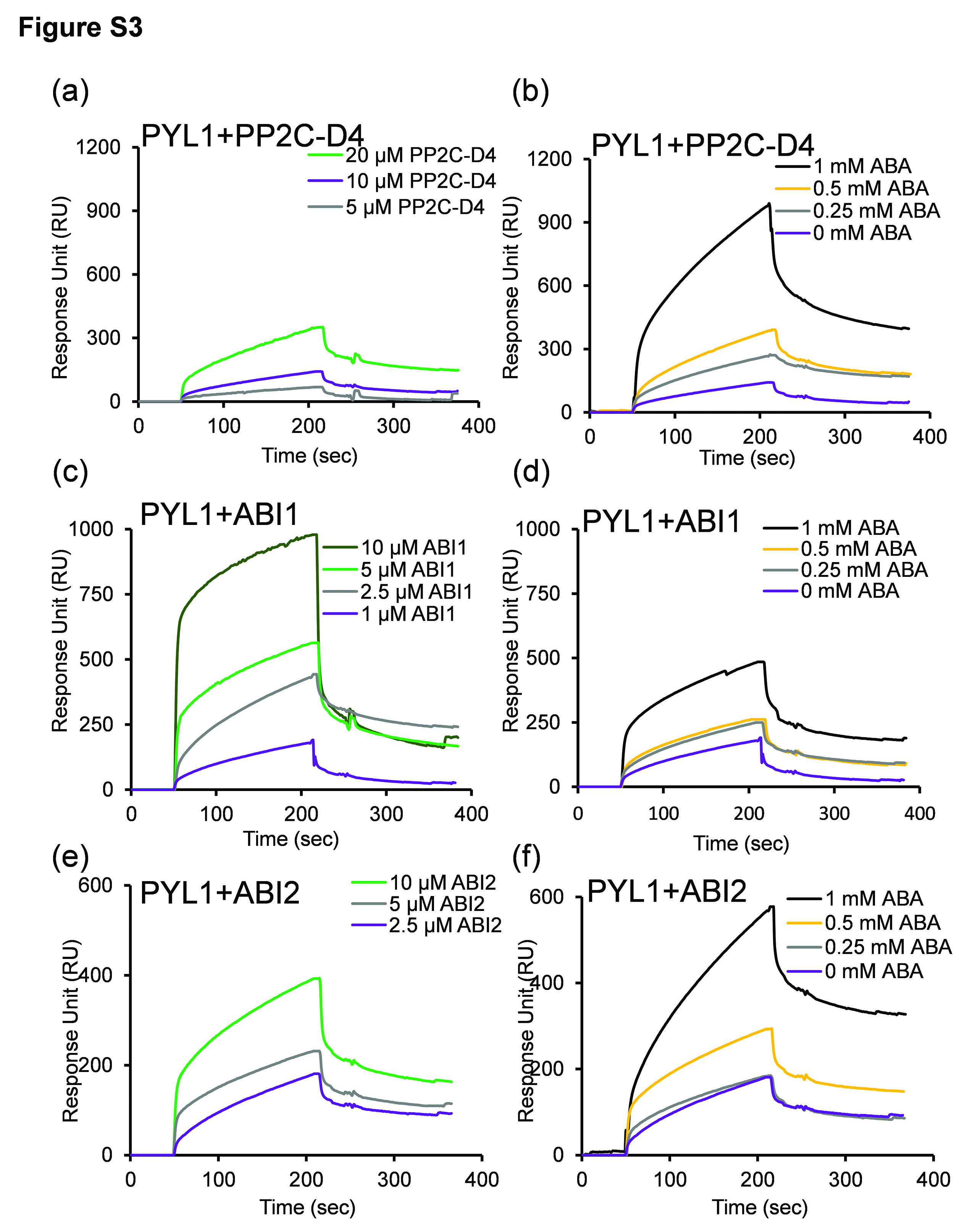

Supplement: Supplementary file 3 [file PLD3-1-e00020-s002.tif]

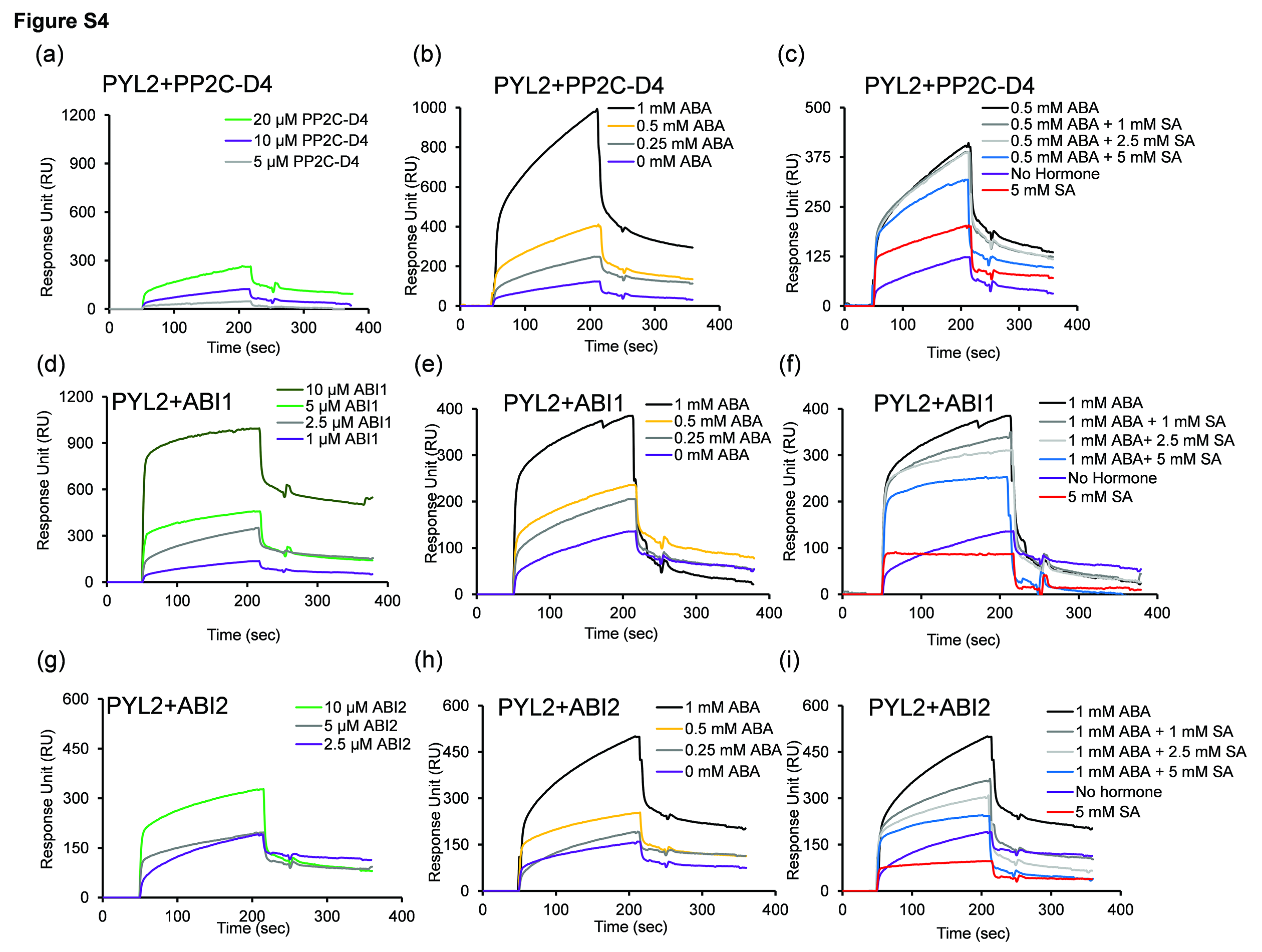

Supplement: Supplementary file 4 [file PLD3-1-e00020-s003.tif]

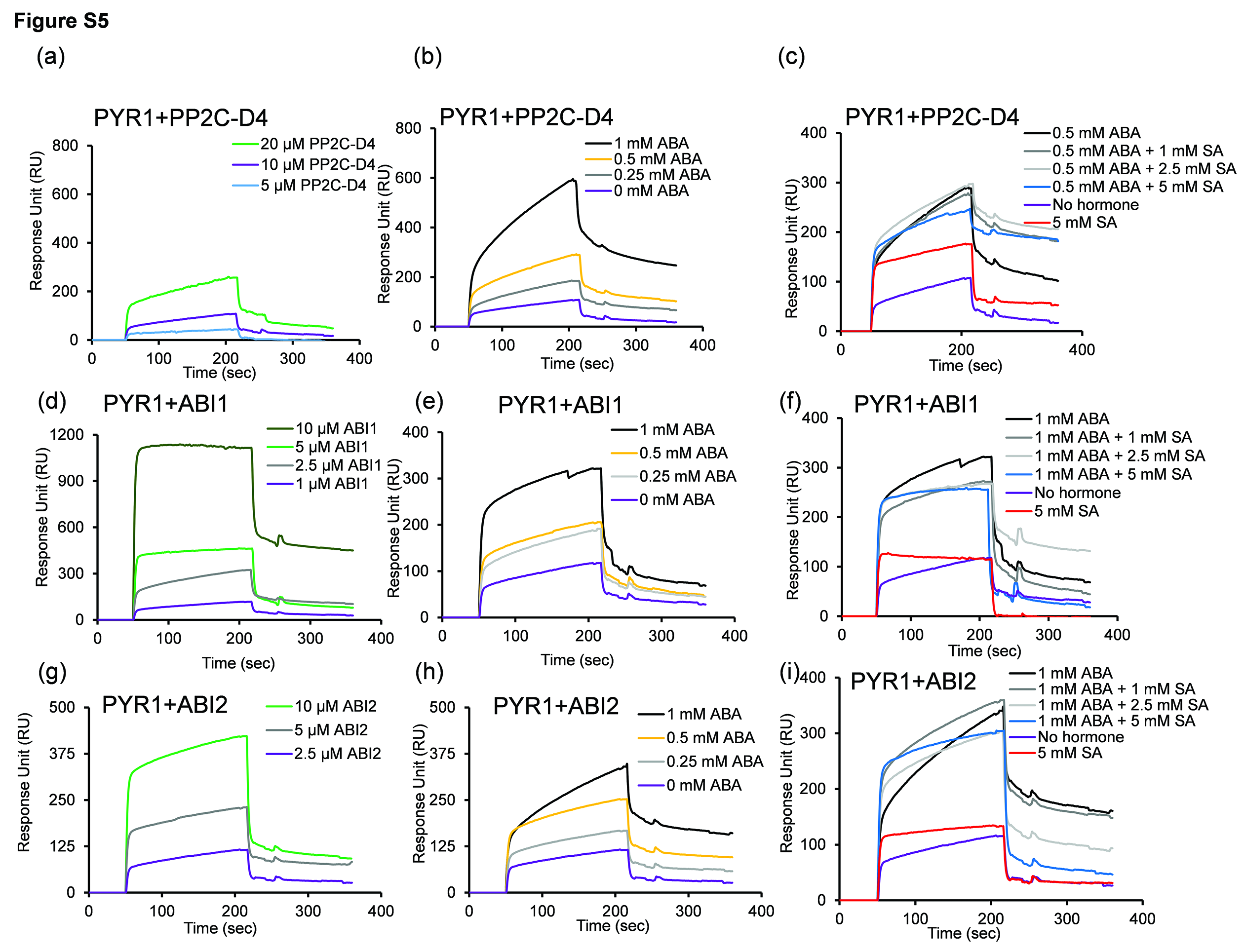

Supplement: Supplementary file 5 [file PLD3-1-e00020-s004.tif]

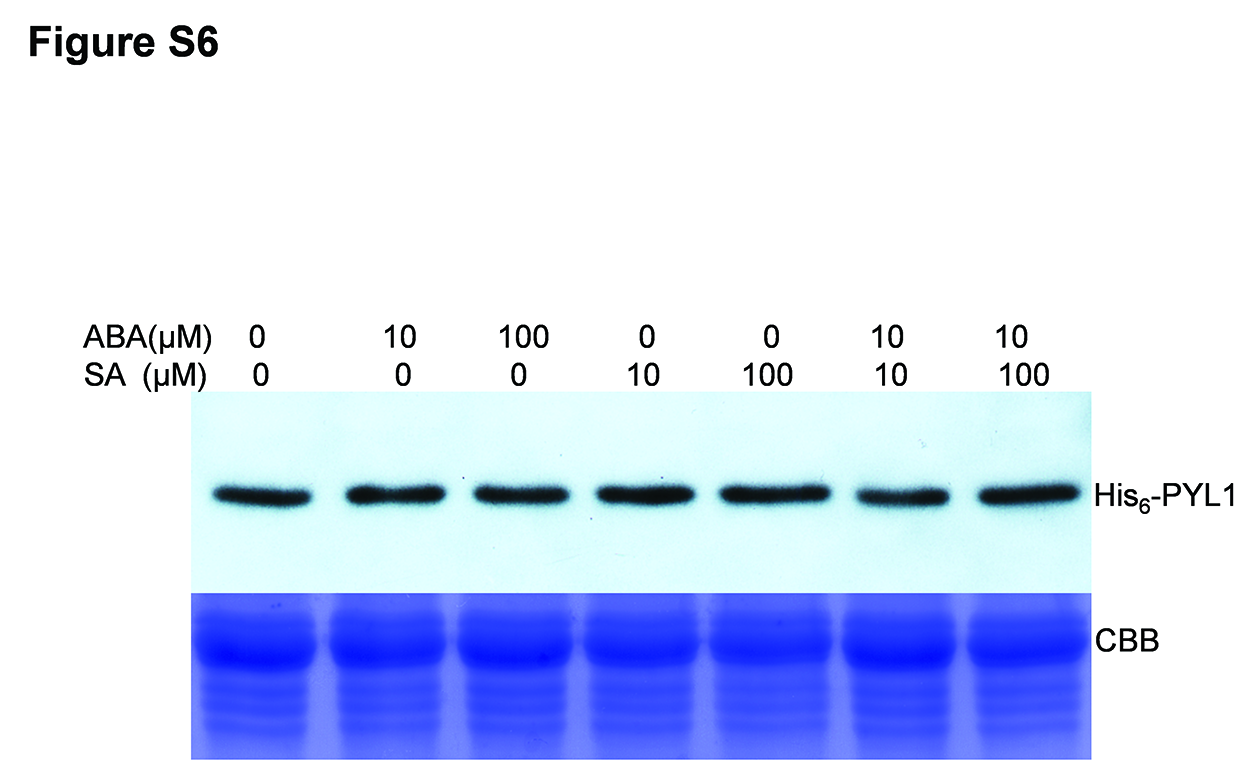

Supplement: Supplementary file 6 [file PLD3-1-e00020-s005.tif]

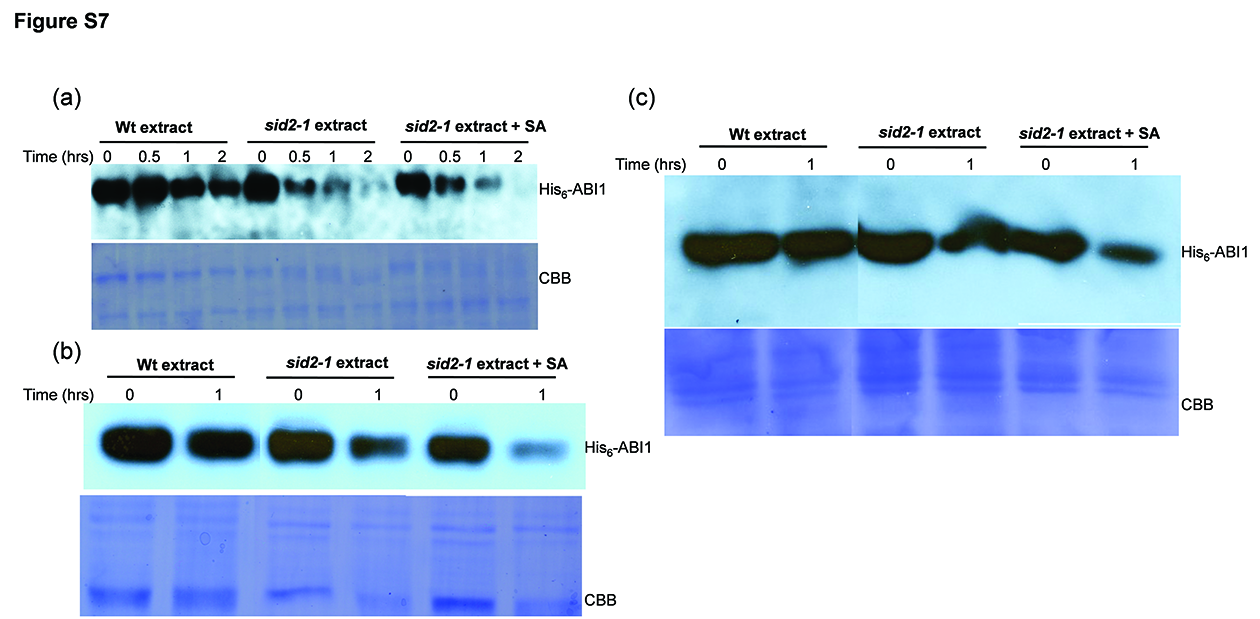

Supplement: Supplementary file 7 [file PLD3-1-e00020-s006.tif]
