# Supplementary material for: Members of the abscisic acid co‐receptor PP2C protein family mediate salicylic acid–abscisic acid crosstalk
Source: Plant Direct. 2017 Nov 6;1(5):e00020. doi: 10.1002/pld3.20 (PMC6508495; doi:10.1002/pld3.20)
Supplement: Supplementary file 8 [file PLD3-1-e00020-s008.pdf]

Table S1: List of primers used in the experiments. "q" indicates primers used for qPCR analysis

ABI1-F	GGA CATATG ATGGAGGAAGTATCTCCGGCG
ABI1-R	GGA GGATCC TCAGTTCAAGGGTTTGCTCTTG
ABI2-F	GGA CATATG ATGGACGAAGTTTCTCCTGCAG
ABI2-R	GGA GAGCTC TCAATTCAAGGATTTGCTCTTG
PP2C-D4-F	GGACATATGATGGTATCTACAACATTTAGG
PP2C-D4-R	GGAGAGCTCCTATAAAACGGGATTATGGGC
HAB1-F	GGA GGATCC ATGGAGGAGATGACTCCCGCAG
HAB1-R	GGA GAGCTC TCAGGTTCTGGTCTTGAACTTTC
qABI1-F	CCATGGCGGTTCTCAGGTAG
qABI1-R	CCACGTATCACCATCGCAGA
qABI2-F	CCATGGCGGTTCTCAGGTAG
qABI2-R	ACCGTCACAAAACTCCGGTT
qPP2CD-F	ATCATGAGAGCGGAACCGAC
qPP2CD-R	ACTTCCGAGCCACACCATTG
qAREB2-F	GCCAATCATGCCTAAGCAGC
qAREB2-R	CTCCACGGTGTAAGCCTGTT
qRD29A-F	GCACCAGGCGTAACAGGTAA
qED29A-R	GCAGAGAGACCGGAGTGTTC
