# Supplementary material for: Members of the abscisic acid co‐receptor PP2C protein family mediate salicylic acid–abscisic acid crosstalk
Source: Plant Direct. 2017 Nov 6;1(5):e00020. doi: 10.1002/pld3.20 (PMC6508495; doi:10.1002/pld3.20)
Supplement: Supplementary file 9 [file PLD3-1-e00020-s009.docx]

**Supporting Information legends**

**Figure S1: HAB1, PP2A and members of the PYR/PYL/RCAR and SnRK2 families are not SABPs** (a-c) Sensorgrams obtained using a 3AESA-immobilized SPR sensor chip with 5 μM of His_6_-HAB1 (At1g72770), His_6_-PP2A (At1g25490), or His_6_-SUMO-SnRK2.2 (At3g50500) in the absence or presence of 2 mM ABA. Signals detected from a mock-coupled control chip were subtracted. (d) Binding of [^3^H]SA (200 nM) by 200 ng/µl SnRK2.2, 2.3, 2.6, PYL1, PYL2, or PYR1 was determined by size-exclusion chromatography. Chromatography with [^3^H]SA in the absence of protein served as negative control.

**Figure S2: SA-binding activities of PP2C-D4 is enhanced by ABA.** Sensorgrams obtained with recombinant, purified 1 μM of His_6_-tagged PP2C-D4 using a 3AESA-immobilized sensor chip in the absence or in the presence of 0.5 or 2 mM ABA. Signals detected from a mock-coupled control chip were subtracted. The experiments was independently repeated at least twice.

**Figure S3: Effect of ABA on the interactions of PP2Cs with PYL1.** (a, c, e) Sensorgrams obtained with the indicated concentrations of recombinant, purified His_6_- tagged PP2C-D4, ABI1, or ABI2 using a His_6_- SUMO-tagged PYL1-immobilized sensor chip**.** (b, d, f) Dose-dependent effect of ABA on the interactions between 10 µM PP2C-D4, 1 µM ABI1, or 2.5 µM ABI2 and His_6_- SUMO-tagged PYL1 immobilized on the sensor chip. Signals detected from a mock-coupled control chip were subtracted. The experiments was independently repeated at least twice.

**Figure S4: SA disrupts the ABA-enhanced interactions between PP2Cs (PP2C-D4, ABI1, and ABI2) and the ABA receptor PYL2.** (a, d, g) Sensorgrams obtained using a His_6_-SUMO-tagged PYL2-immobilized sensor chip and the indicated concentrations of recombinant, purified His_6_- tagged PP2C-D4, ABI1, or ABI2**.** (b, e, h) Dose-dependent effect of ABA on the interactions of 10 µM PP2C-D4, 1 µM ABI1, or 2.5 µM ABI2 with His_6_-SUMO-tagged PYL2-immobilized sensor chip. (c, f, i) Sensorgrams obtained with 10 μM of the His_6_- tagged PP2C-D4, 1 µM ABI1, or 2.5 µM ABI2 using a His_6_-SUMO-tagged PYL2-immobilized sensor chip in the absence or presence of the indicated concentrations of ABA, SA or ABA plus SA. Signals detected from a mock-coupled control chip were subtracted. The experiments was independently repeated at least twice.

**Figure S5: SA disrupts the ABA-enhanced interactions between PP2Cs (PP2C-D4, ABI1, and ABI2) and ABA receptor PYR1.** (a, d, g) Sensorgrams obtained using a His_6_- SUMO-tagged PYR1-immobilized sensor chip and the indicated concentrations of recombinant, purified His_6_- tagged PP2C-D4, ABI1, or ABI2**.** (b, e, h) ABA dose-dependent effect on the interactions of 10 µM PP2C-D4, 1 µM ABI1, or 2.5 µM ABI2 with His_6_-SUMO-tagged PYR1-immobilized sensor chip. (c, f, i) Sensorgrams obtained with recombinant 10 μM of the His_6_- tagged PP2C-D4, 1 µM ABI1 or 2.5 µM ABI2 using a His_6_- SUMO-tagged PYR1-immobilized sensor chip in the absence or presence of the indicated concentrations of ABA, SA, or ABA plus SA. Signals detected from a mock-coupled control chip were subtracted. The experiments was independently repeated at least twice.

**Figure S6: ABA and SA alone or in combination do not affect turnover of PYL1.** Cell-free degradation assay using approximately 100 µg of total protein extracts prepared from ten-day-old Arabidopsis seedlings supplemented with 500 ng of His_6_-Sumo-tagged PYL1 and indicated concentrations of ABA, SA, or ABA+SA. The degradation assay was carried out at 30^0^ C for 3 hrs. Proteins were detected by immunoblotting using an α-His_6_-HRP antibody and Coomassie brilliant blue (CBB) staining of the gel served as a loading control. The experiments was independently repeated twice.

**Figure S7: SA does not affect the rate of ABI1 degradation in *sid2-1* plants.** (a) Cell-free degradation assay using 500 ng of His_6_- tagged ABI1 and approximately 100 µg of Arabidopsis protein extracts from Wt or *sid2-1* mutant plants in the absence or presence of 10 µM SA that was added directly to the reaction mix. His_6_- tagged ABI1 was detected in samples harvested at the indicated times by immunoblotting using a α-His_6_-HRP antibody. (b) Cell-free degradation assay using 500 ng of His_6_-tagged ABI1 and approximately 100 µg of Arabidopsis protein extracts from ten-day-old Wt or *sid2-1* mutant plants that were sprayed with water or 10 µM SA three hours prior to extract preparation. His_6_- tagged ABI1 was detected in samples harvested at the indicated times by immunoblotting using a α-His_6_-HRP antibody. (c) Cell-free degradation assay using Arabidopsis protein extracts from ten-day-old wild-type (Wt) or SA-deficient *sid2-1* mutant plants grown in MS medium in the absence or presence of 10 µM SA and supplemented with 500 ng of His_6_-tagged ABI1. His_6_- tagged ABI1 was detected in samples harvested at the indicated times by immunoblotting using an α-His_6_-HRP antibody**.** For b & c, all lanes are from the same experiment; some lanes unrelated to this study were removed and lanes were then merged for clarity of presentation. The experiments was independently repeated twice.
